# Integrated multi-omics analysis suggests the involvement of PI3K-Akt/p21 signaling in the anti-colorectal cancer effects of *Diaphragma Juglandis Fructus* extract

**DOI:** 10.3389/fphar.2026.1833123

**Published:** 2026-06-01

**Authors:** Yingjuan Yang, Yifeng Xu, Haotian Cheng

**Affiliations:** School of Life Science, Shanxi University, Taiyuan, Shanxi, China

**Keywords:** apoptosis, cell cycle arrest, colorectal cancer, *Diaphragma Juglandis Fructus*, PI3K-Akt signaling, proteomics

## Abstract

**Background:**

Colorectal cancer (CRC) remains a leading cause of cancer-related mortality worldwide, underscoring the need for more effective and well-tolerated therapeutic strategies. *Diaphragma Juglandis Fructus* (DJF), a by-product of walnut processing used in traditional medicine, is rich in polyphenolic compounds, yet its potential effects on CRC have not been fully characterized. This study aimed to evaluate the anti-colorectal cancer activity of the ethanol extract of DJF (EEDJF) and explore the underlying molecular mechanisms using an integrated multi-omics strategy.

**Methods:**

Network pharmacology, molecular docking, and label-free proteomics were combined with cell-based assays. Functional relevance of AKT signaling was evaluated using siRNA knockdown, MK2206, and SC79.

**Results:**

Fifty-three putative bioactive compounds and 154 CRC-related targets were identified, with AKT1, EGFR, and SRC emerging as candidate hub proteins. EEDJF significantly inhibited proliferation in HCT116 and SW480 colorectal cancer cells, induced apoptosis, and promoted G1/S cell cycle arrest. In contrast, EEDJF showed minimal cytotoxicity toward HaCaT epithelial cells under the tested conditions. Proteomic and enrichment analyses consistently highlighted the PI3K-Akt-related signaling among the most significantly associated pathways. Modulation of AKT activity altered the cellular response to EEDJF, supporting the involvement of this pathway.

**Conclusion:**

EEDJF exerts anti-colorectal cancer effects *in vitro*, potentially associated with regulation of PI3K-Akt/p21 signaling. These findings provide a basis for further studies on the bioactive constituents, pharmacological mechanisms, and *in vivo* efficacy of DJF-derived preparations.

## 1 Introduction

Colorectal cancer (CRC) ranks as the third most frequently diagnosed malignancy, accounting for 9.6% of global cancer incidence, and the second leading cause of cancer-related mortality worldwide, responsible for 9.3% of all cancer deaths (Bray et al., 2024). The burden is particularly substantial in China, where colorectal cancer has shown a rapidly increasing incidence and mortality in recent years (Diao et al., 2025). Although advances in surgical intervention, chemotherapy, and targeted therapies have improved clinical outcomes, recurrence, metastasis, and therapeutic resistance remain major challenges. Therefore, the identification of effective and low-toxicity therapeutic agents remains an urgent priority.

Natural products have historically provided a rich source of bioactive compounds for anticancer drug discovery. Due to their structural diversity and ability to modulate multiple molecular targets, plant-derived compounds have demonstrated considerable potential in cancer therapy (Atanasov et al., 2021). Increasing evidence suggests that phytochemicals can interfere with key hallmarks of cancer, including sustained proliferation, resistance to apoptosis, and aberrant signaling through regulation of critical oncogenic pathways (Hanahan, 2022). For instance, *Podocarpus elatus* extracts suppress CRC cell growth by inducing S-phase arrest and autophagy (Akter et al., 2018), whereas *Olea europaea* leaf and *Annona muricata* extracts exert antioxidant and pro-apoptotic effects through mitochondrial and PI3K-Akt pathway modulation (Maalej et al., 2017; Moghadamtousi et al., 2014). These findings highlight the importance of natural products as a valuable resource for identifying novel anticancer agents targeting key signaling networks.

*Diaphragma Juglandis Fructus* (DJF), derived from the walnut (*Juglans regia L.*), is an underutilized by-product rich in polyphenols, flavonoids, and saponins. Previous phytochemical analyses have identified multiple bioactive constituents in DJF, including quercetin and ellagic acid, which exhibit antioxidant, anti-inflammatory, and potential anticancer activities (Liu et al., 2019; Hu et al., 2020). Notably, these compounds have been reported to suppress tumor growth and metastasis through modulation of pathways such as PI3K-Akt, mitochondrial signaling, and oxidative stress regulation (Lu et al., 2023). Despite these advances, the integrated molecular mechanisms and target networks underlying the anticancer effects of DJF, particularly its ethanol extract (EEDJF), remain largely unclear.

Among the oncogenic pathways implicated in CRC, the PI3K-Akt signaling pathway plays a central role in regulating cell proliferation, survival, metabolism, and cell cycle progression. Aberrant activation of this pathway is frequently observed in CRC and is associated with tumor progression, metastasis, and resistance to therapy. Therefore, targeting PI3K-Akt signaling represents a promising strategy for CRC treatment.

In the present study, we employed an integrated multi-omics approach combining network pharmacology, molecular docking, and label-free quantitative proteomics to systematically elucidate the bioactive compounds, molecular targets, and signaling pathways associated with EEDJF. Furthermore, mechanistic validation was performed using AKT-targeted siRNA, the inhibitor MK2206, and the activator SC79 to confirm the functional role of PI3K-Akt signaling in mediating EEDJF-induced apoptosis and cell cycle arrest. By integrating computational prediction with experimental validation, this study provides a comprehensive mechanistic framework for understanding the anti-CRC activity of EEDJF and highlights its potential as a source of bioactive compounds for anticancer therapy.

## 2 Materials and methods

### 2.1 Chemicals and reagents

HCT116 and SW480 cells were obtained from the Cell Bank of the Chinese Academy of Sciences (Shanghai, China). Dulbecco’s Modified Eagle Medium (DMEM) and fetal bovine serum (FBS) were purchased from Tianhang Biotechnology (Zhejiang, China). MTT, penicillin/streptomycin, and DMSO were obtained from Solarbio (Beijing, China). Protein quantification and lysis reagents were from Boser Biotechnology (Wuhan, China). The cell cycle analysis kit was from Abbkine (Wuhan, China). Antibodies were purchased as follows: p-PI3K from Bioss (Beijing, China); PI3K and p21 from Proteintech (Wuhan, China); p-Akt, Cyclin D1, and CDK4 from Wanleibio (Shenyang, China); and total AKT from Cell Signaling Technology (MA, USA). Flow cytometry equipment was supplied by Agilent Technologies (Beijing, China).

### 2.2 Cell culture and cell viability assay

HCT116 and SW480 cells were cultured in DMEM with 10% FBS and 1% penicillin-streptomycin at 37 °C in a 5% CO_2_ incubator. For MTT assays, cells were seeded in 96-well plates and treated with EEDJF (10–320 μg/mL) for 24 h. MTT (10 μL, 5 mg/mL) was added for 4 h, followed by the addition of 150 μL DMSO. Absorbance was measured at 490 nm.

### 2.3 Preparation of EEDJF

*Diaphragma Juglandis Fructus* (DJF) was obtained from Urumqi, Xinjiang, China. The samples were dried, pulverized, and sieved through a 60-mesh screen. One gram of DJF powder was extracted with 50% ethanol (solid-to-liquid ratio 1:62, g/mL) using ultrasonic-assisted extraction at 71 °C for 50 min. The extract was centrifuged at 3,000 × g for 15 min to collect the supernatant, and the process was repeated three times. The combined supernatants were evaporated at 45 °C and lyophilized to obtain the ethanol extract (EEDJF), which was stored at −20 °C in the dark until use.

### 2.4 Component identification via HPLC-MS/MS

The active constituents of EEDJF were characterized using an Agilent 1,290 Infinity UHPLC system equipped with a C18 column (40 °C). The mobile phase consisted of solvent A (water containing 25 mM ammonium acetate and 0.5% formic acid) and solvent B (methanol). The gradient program was: 0–0.5 min, 5% B; 0.5–10.0 min, 5%–100% B; 10.0–12.0 min, 100% B; 12.0–12.1 min, 100%–5% B; and 12.1–16.0 min, 5% B. The flow rate was maintained at 0.4 mL/min, and detection was performed in both positive and negative ion modes with the sample tray maintained at 4 °C.

### 2.5 Network pharmacology analysis

#### 2.5.1 Identification of active compounds and potential targets

Bioactive compounds in EEDJF were retrieved using the Traditional Chinese Medicine Systems Pharmacology Database (TCMSP, https://old.tcmsp-e.com/tcmsp.php) with oral bioavailability (OB) ≥ 30% and drug-likeness (DL) ≥ 0.18. The compound structures (SDF format) were downloaded from PubChem (https://pubchem.ncbi.nlm.nih.gov) and imported into PharmMapper (http://www.lilab-ecust.cn/pharmmapper) for target prediction (Norm Fit >0.5). The identified targets were standardized using Uniprot (https://www.uniprot.org).

#### 2.5.2 Identification of CRC-related targets

Colorectal cancer-related targets were obtained from the GeneCards (https://www.genecards.org/), TTD (https://db.idrblab.net/ttd/), PharmGkb (https://www.pharmgkb.org/), DrugBank (https://go.drugbank.com/), and OMIM (https://omim.org/) databases using “colon cancer”, “colon tumor”, “rectal cancer”, and “rectal tumor” as search terms. Targets with GeneCards scores ≥10 were retained. All retrieved targets were merged, and duplicates were removed.

#### 2.5.3 Construction of compound–target and PPI networks

Common targets shared by EEDJF and CRC were identified using R 4.2.3 and visualized in Cytoscape 3.8.0. The protein - protein interaction (PPI) network was constructed using STRING (https://string-db.org/; species: *Homo sapiens*; confidence score ≥0.9; isolated nodes hidden), and topological parameters were analyzed with Network Analyzer.

#### 2.5.4 GO and KEGG enrichment analysis

Gene Ontology (GO) and Kyoto Encyclopedia of Genes and Genomes (KEGG) pathway enrichment analyses were conducted using SangerBox 3.0 (Shen et al., 2022). The GO terms were categorized into biological process (BP), cellular component (CC), and molecular function (MF) domains. The top enriched pathways were ranked according to p-value significance.

### 2.6 Molecular docking

Ligand structures were obtained from PubChem and optimized using Chem3D, while receptor protein structures were retrieved from the Protein Data Bank (PDB, https://www.rcsb.org/). Docking simulations were performed using AutoDock Vina (rigid receptor-flexible ligand mode). Binding energies <0 kcal/mol indicated spontaneous binding interactions.

### 2.7 Multi-omics research: 4D label-free quantitative proteomics

#### 2.7.1 Protein preparation

HCT116 cells were treated with EEDJF (125 μg/mL) or DMSO for 24 h using three independent biological replicates per group. Cells were lysed in SDT buffer (4% SDS, 100 mM Tris-HCl, 1 mM DTT, pH 7.6). Protein concentrations were determined by the BCA assay (Bio-Rad, USA). Proteins were digested using the filter-aided sample preparation (FASP) method (Wiśniewski et al., 2009). Peptides were desalted using C18 cartridges (Empore SPE) and vacuum-dried before reconstitution in 0.1% formic acid.

#### 2.7.2 LC-MS/MS analysis

Peptide samples were separated on a Thermo Acclaim PepMap100 column and analyzed using an EASY-nLC system coupled to a timsTOF Pro MS under PASEF mode. The parameters were as follows: scan range 100–1700 m/z; ion source voltage 1.5 kV; cycle time 1.17 s; and exclusion time 24 s. Each biological replicate was analyzed independently by LC-MS/MS.

#### 2.7.3 Protein identification and annotation

Raw data were processed with MaxQuant 1.6.14 (enzyme: trypsin; max 2 missed cleavages; FDR<1%). Subcellular localization was predicted using CELLO (http://cello.life.nctu.edu.tw/). Functional annotation was performed using Blast2GO, and KEGG enrichment with KAAS.

#### 2.7.4 PPI network analysis

Protein–protein interactions were retrieved from the IntAct database (http://www.ebi.ac.uk/intact/main.xhtml) and analyzed using CytoScape 3.2.1.

### 2.8 Cell transfection

HCT116 cells were transfected with siAKT using TransIntro® EL reagent (250 pmol siRNA per well). After 4 h, the medium was replaced, and cells were cultured for 24 h before analysis.

### 2.9 Western blot analysis

Cells were lysed in PMSF-containing buffer, and protein extracts (12,000 rpm, 4 °C, 20 min) were separated by SDS-PAGE and transferred onto PVDF membranes (Millipore, USA). Membranes were blocked with 5% skim milk for 1 h at room temperature, then incubated overnight with primary antibodies at 4 °C, followed by IRDye-conjugated secondary antibodies for 2 h in the dark. Protein bands were visualized with a dual-color infrared imaging system (LI-COR odyssey, USA). Band intensities were quantified using ImageJ software and normalized to β-actin. All Western blot analyses were repeated in three independent biological experiments.

### 2.10 Cell cycle analysis

HCT116 cells (2 × 10^5^ cells/mL) were fixed in 70% cold ethanol overnight at 4 °C and stained with propidium iodide (PI) for 30 min. The cell cycle distribution was analyzed by flow cytometry (NovoExpress 1.5.6, Agilent, USA).

### 2.11 Statistical analysis

All experiments were independently performed at least three times unless otherwise specified. Data are presented as means ± SD from biological replicates. Statistical significance was determined using GraphPad 9.0.0 (GraphPad Inc., USA). Comparisons between two groups were analyzed using Student’s t-test, while multiple-group comparisons were evaluated by one-way ANOVA. Differences were considered statistically significant at p < 0.05.

## 3 Results

### 3.1 Network pharmacology analysis

#### 3.1.1 Identification of main components and potential targets of EEDJF

Natural products have long served as valuable sources of anticancer agents due to their chemical diversity, multitarget activity, and favorable safety profiles. *Diaphragma Juglandis Fructus* (DJF), a traditional Chinese medicinal by-product derived from walnut septum, contains abundant polyphenols and flavonoids with reported antioxidant and anti-inflammatory activities.

**FIGURE 1:**
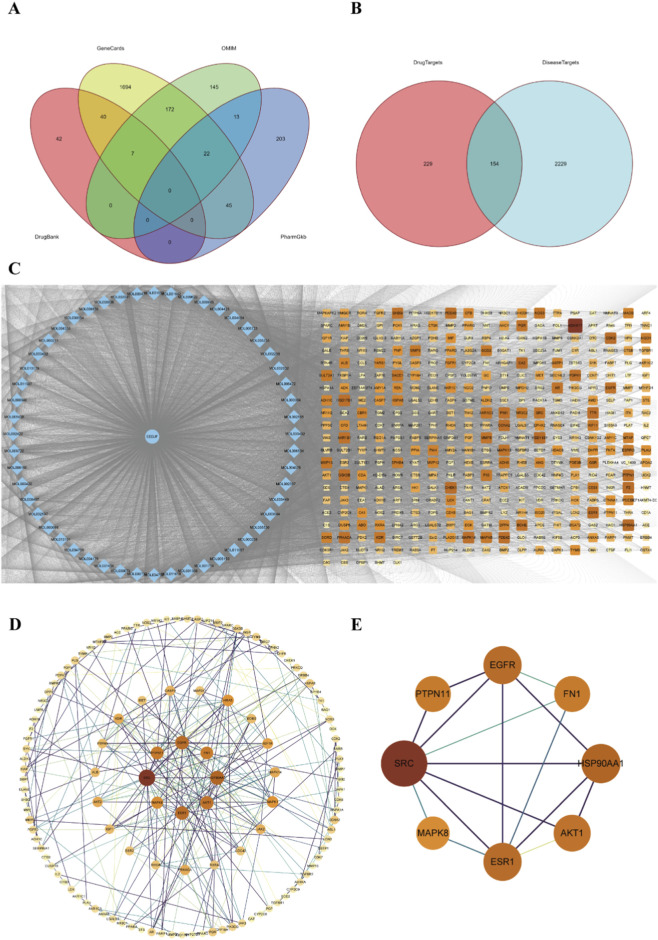
Network pharmacology workflow and target identification of EEDJF against colorectal cancer (CRC). **(A)** Identification of CRC-related targets from multiple databases. **(B)** Venn diagram showing intersecting targets between EEDJF and CRC. **(C)** Compound-target interaction network of EEDJF. **(D)** Protein-protein interaction (PPI) network of common targets generated using the STRING database. **(E)** Core hub genes identified through topological analysis of the PPI network.

In this study, 2,383 CRC-related targets were retrieved from multiple databases (Figure 1A). Based on the criteria of oral bioavailability (OB ≥ 30%) and drug-likeness (DL ≥ 0.18), 53 active constituents of the EEDJF were identified by LC-MS/MS (Table 1). PharmMapper prediction further identified 154 overlapping targets shared by EEDJF components and CRC-associated genes (Figure 1B), suggesting a multitarget pharmacological profile.

**TABLE 1:** The main active ingredients of EEDJF.

MOL ID	Mol name	OB (%)	DL	Rt (min)
MOL006504	(-) -Catechin gallate	53.57	0.75	3.795
MOL001714	(-) -Podophyllotoxin	59.94	0.86	5.101
MOL000073	(+) -epicatechin	48.96	0.24	10.731
MOL005190	(2S,3S)-3,4',5,7-tetrahydroxyflavanone	71.79	0.24	5.293
MOL001368	3-p-Coumaroylquinic acid	37.63	0.29	2.602
MOL006160	Alizarin	32.67	0.19	5.872
MOL000471	Aloe-emodin	83.38	0.24	8.763
MOL004355	Alpha-spinasterol	42.98	0.76	13.286
MOL006472	Aurantio-obtusin	31.55	0.37	6.844
MOL001454	Berberine	36.86	0.78	5.68
MOL000211	Betulinic acid	55.38	0.78	11.483
MOL000940	Bisdemethoxycurcumin	77.38	0.26	5.222
MOL005438	Campesterol	37.58	0.71	13.647
MOL003044	Chrysoeriol	35.85	0.27	5.522
MOL000492	Cianidanol	54.83	0.24	3.36
MOL000258	Dehydrodiisoeugenol	56.84	0.29	5.63
MOL003402	Demethylwedelolactone	72.13	0.43	6.575
MOL008845	Deoxycholic acid	40.72	0.68	11.127
MOL004576	Dihydroquercetin	57.84	0.27	3.841
MOL002032	Dioctyl phthalate	40.59	0.40	12.402
MOL001002	Ellagic acid	43.06	0.43	6.063
MOL005235	Embelin	37.72	0.18	9.46
MOL005190	Eriodictyol	71.79	0.24	5.89
MOL002597	Euphol	42.12	0.75	13.71
MOL013179	Fisetin	52.60	0.24	6.245
MOL011616	Fortunellin	35.65	0.74	4.673
MOL009622	Fucosterol	43.78	0.76	12.507
MOL011587	Ginkgolide C	48.33	0.73	3.738
MOL006733	Ginsenoside F2	37.03	0.25	13.249
MOL002341	Hesperetin	70.31	0.27	6.418
MOL008487	Hirsutine	34.44	0.43	6.093
MOL005530	Hydroxygenkwanin	36.47	0.27	7.018
MOL004425	Icariin	41.58	0.61	5.178
MOL001942	Isoimperatorin	45.46	0.23	7.029
MOL000354	Isorhamnetin	49.60	0.31	6.849
MOL002422	Isotalatizidine	50.82	0.73	10.565
MOL004564	Kaempferide	73.41	0.27	6.871
MOL000422	Kaempferol	41.88	0.24	6.735
MOL002397	Karakoline	51.73	0.73	10.699
MOL009722	Leucovorin	31.79	0.74	1.895
MOL000006	Luteolin	36.16	0.25	6.338
MOL011597	Luteolin-4'-o-glucoside	41.97	0.79	4.976
MOL002565	Medicarpin	49.22	0.34	5.534
MOL000737	Morin	46.23	0.27	5.161
MOL004759	Napelline	34.48	0.72	9.636
MOL004792	Nodakenin	57.12	0.69	4.979
MOL000004	Procyanidin B1	67.87	0.66	3.888
MOL000098	Quercetin	46.43	0.28	6.212
MOL002268	Rhein	47.07	0.28	7.815
MOL013101	Rutarin	70.10	0.20	3.376
MOL000449	Stigmasterol	43.83	0.76	13.279
MOL006859	Triamcinolone	35.42	0.63	10.673
MOL004179	Vernolic acid	37.63	0.19	10.167

#### 3.1.2 Construction of a component-target and PPI networks

A comprehensive component-target interaction network was generated using Cytoscape, consisting of 438 nodes and 14,980 edges (Figure 1C). The PPI network constructed from STRING contained 124 nodes and 276 edges (Figure 1D). Topological analysis identified eight hub proteins with high centrality values - AKT1, ESR1, SRC, MAPK8, HSP90AA1, EGFR, FN1 and PTPN11 (Figure 1E).

#### 3.1.3 Functional enrichment analysis

**FIGURE 2:**
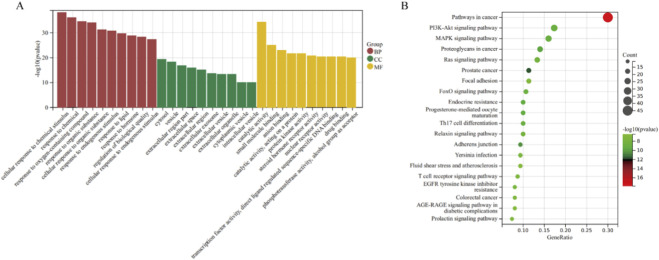
GO and KEGG enrichment analyses of potential targets of EEDJF against colorectal cancer (CRC). **(A)** Top 10 enriched Gene Ontology (GO) terms, including biological processes, molecular functions, and cellular components. **(B)** Top 20 significantly enriched Kyoto Encyclopedia of Genes and Genomes (KEGG) pathways associated with EEDJF activity in CRC.

GO and KEGG enrichment analyses were performed on the 154 putative targets. The enriched GO terms and KEGG pathways are presented in Figure 2. Notably, PI3K-Akt, MAPK, Ras, and cancer-related pathways were significantly enriched, indicating that EEDJF exerts anticancer effects through coordinated regulation of multiple signaling networks. Among these, the PI3K-Akt pathway was selected for further validation because of its established role in CRC progression.

#### 3.1.4 Molecular docking

**FIGURE 3:**
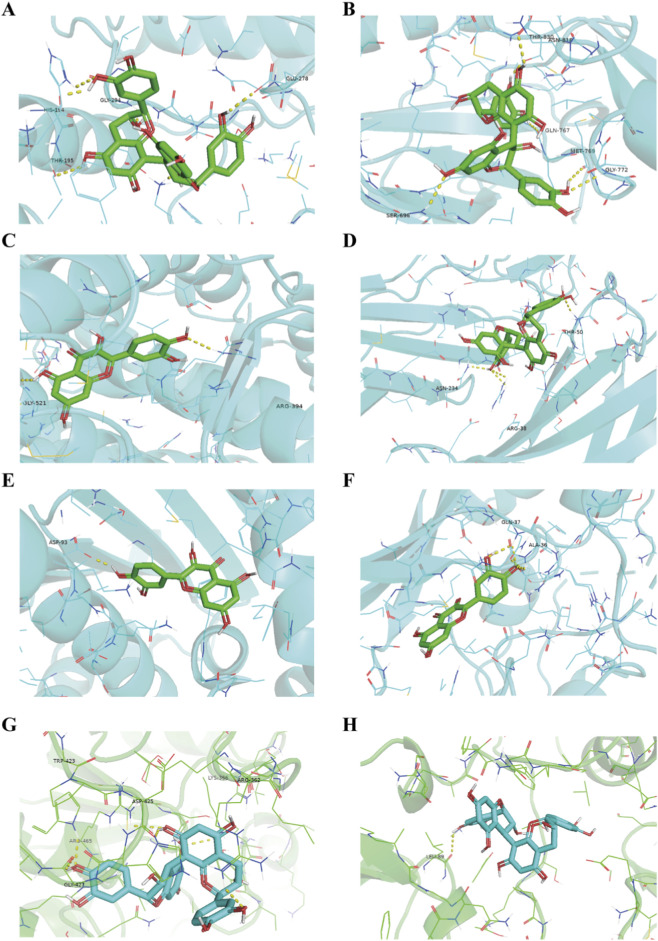
Molecular docking analysis of major EEDJF compounds with core protein targets. Representative docking models illustrating the interactions between key bioactive compounds and hub proteins: **(A)** AKT1-Procyanidin B1, **(B)** EGFR-Procyanidin B1, **(C)** ESR1-Quercetin, **(D)** FN1-Procyanidin B1, **(E)** HSP90AA1-Quercetin, **(F)** MAPK8-Quercetin, **(G)** PTPN11-Procyanidin B1, and **(H)** SRC-Procyanidin B1. Lower binding energy generally indicates a stronger predicted binding affinity.

Molecular docking analysis demonstrated favorable binding affinities between key EEDJF components and core PPI targets (Table 2), with Procyanidin B1 and quercetin showing the strongest interactions (Figure 3), suggesting that these polyphenolic constituents may contribute to the biological activity of EEDJF through direct target engagement.

**TABLE 2:** Molecular docking binding energy (kcal/mol).

Ingredients	Cianidanol	3-p-Coumaroylquinic acid	Quercetin	Procyanidin B1	Dihydroquercetin
AKT1	−7.8	−7.5	−8.5	−9.0	−7.2
HSP90AA1	−8.2	−9.1	−9.1	−9.0	−8.0
PTPN11	−6.8	−6.9	−7.3	−8.1	−7.1
SRC	−8.2	−8.2	−8.8	−9.3	−8.2
MAPK8	−7.8	−7.9	−8.7	−8.1	−7.8
FN1	−6.8	−6.7	−7.1	−7.8	−7.2
EGFR	−7.2	−7.2	−8.0	−8.5	−7.5
ESR1	−7.3	−8.2	−8.8	−8.7	−7.2

### 3.2 Proteomic profiling of EEDJF-treated CRC cells

#### 3.2.1 EEDJF suppresses CRC cell proliferation

**FIGURE 4:**
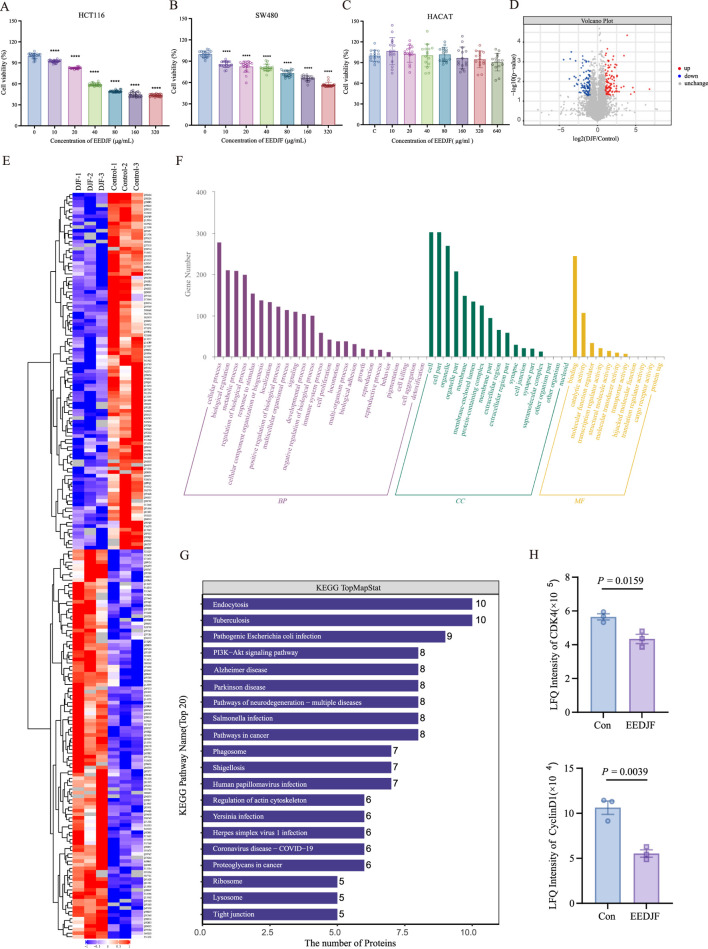
Proteomic profiling of EEDJF-treated colorectal cancer (CRC) cells. **(A–C)** Effects of EEDJF on the viability of HCT116, SW480, and HaCaT cells. **(D)** Volcano plot showing differentially expressed proteins (DEPs) between control and EEDJF-treated groups. **(E)** Hierarchical clustering analysis of DEPs. **(F)** Gene Ontology (GO) enrichment analysis of DEPs. **(G)** Top 20 enriched KEGG pathways associated with DEPs. **(H)** Quantitative analysis of cell cycle-related proteins. Data are presented as mean ± SD from three independent biological experiments. *p < 0.05, **p < 0.01, ***p < 0.001, ****p < 0.0001.

MTT assay revealed that EEDJF inhibited cell viability in HCT116 and SW480 cells in a dose-dependent manner (Figures 4A,B). HCT116 cells were more sensitive (IC_50_ = 125.9 μg/mL) and were therefore selected for subsequent proteomic analysis. To preliminarily evaluate the selectivity of EEDJF, its effects on non-malignant HaCaT epithelial cells were also examined. Under the tested concentrations, EEDJF showed no obvious cytotoxicity toward HaCaT cells (Figure 4C), suggesting a relatively lower inhibitory effect on non-cancerous cells compared with CRC cells.

#### 3.2.2 Identification of differentially expressed proteins

Proteomic profiling was performed using three independent biological replicates per group to ensure reproducibility. Label-free quantitative proteomics yielded 337,894 spectra, including 134,551 valid spectra, 31,075 unique peptides, and 5,303 proteins, of which 5,207 were quantified. Based on fold-change (FC > 2) and p-value (<0.05), 207 DEPs were identified, including 108 upregulated and 99 downregulated proteins (Figure 4D).

#### 3.2.3 Functional annotation and enrichment analysis

Hierarchical clustering and enrichment analyses (Figures 4E–H) indicated that the DEPs were mainly involved in biological regulation, signal transduction, and cell cycle processes. KEGG analysis again highlighted PI3K–Akt signaling.

**FIGURE 5:**
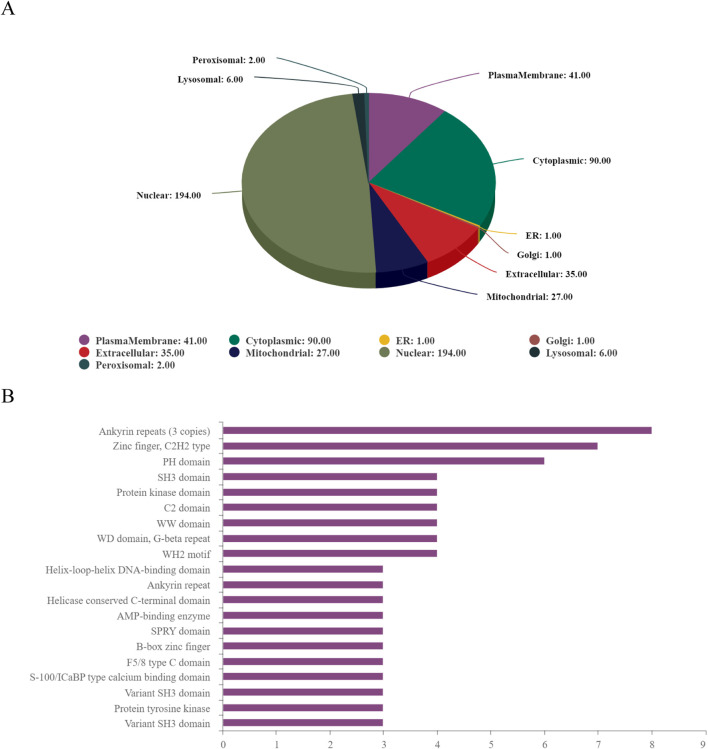
Subcellular localization and protein domain enrichment of differentially expressed proteins (DEPs). **(A)** Subcellular distribution of DEPs across different cellular compartments. **(B)** Enrichment of conserved protein domains identified among DEPs.

Subcellular localization analysis showed that most DEPs were nuclear proteins (Figure 5A), whereas domain enrichment revealed ankyrin repeats, C2H2-type zinc fingers, and PH domains (Figure 5B).

#### 3.2.4 Integrated analysis of proteomics and network pharmacology

Integrated analysis reinforced the PI3K-Akt pathway as the central mechanistic axis underlying EEDJF’s anti-CRC activity. Key regulators, including PI3K, AKT, and downstream effectors, were closely associated with apoptosis and cell cycle regulation. EEDJF treatment significantly upregulated p21 and downregulated Cyclin D1 and CDK4 (Figure 4H), indicating induction of G1/S arrest and apoptotic priming. The consistency among network pharmacology predictions, proteomic profiling, and functional assays strengthen the reliability of the proposed mechanism.

### 3.3 EEDJF induces apoptosis via inhibition of the PI3K-Akt pathway

**FIGURE 6:**
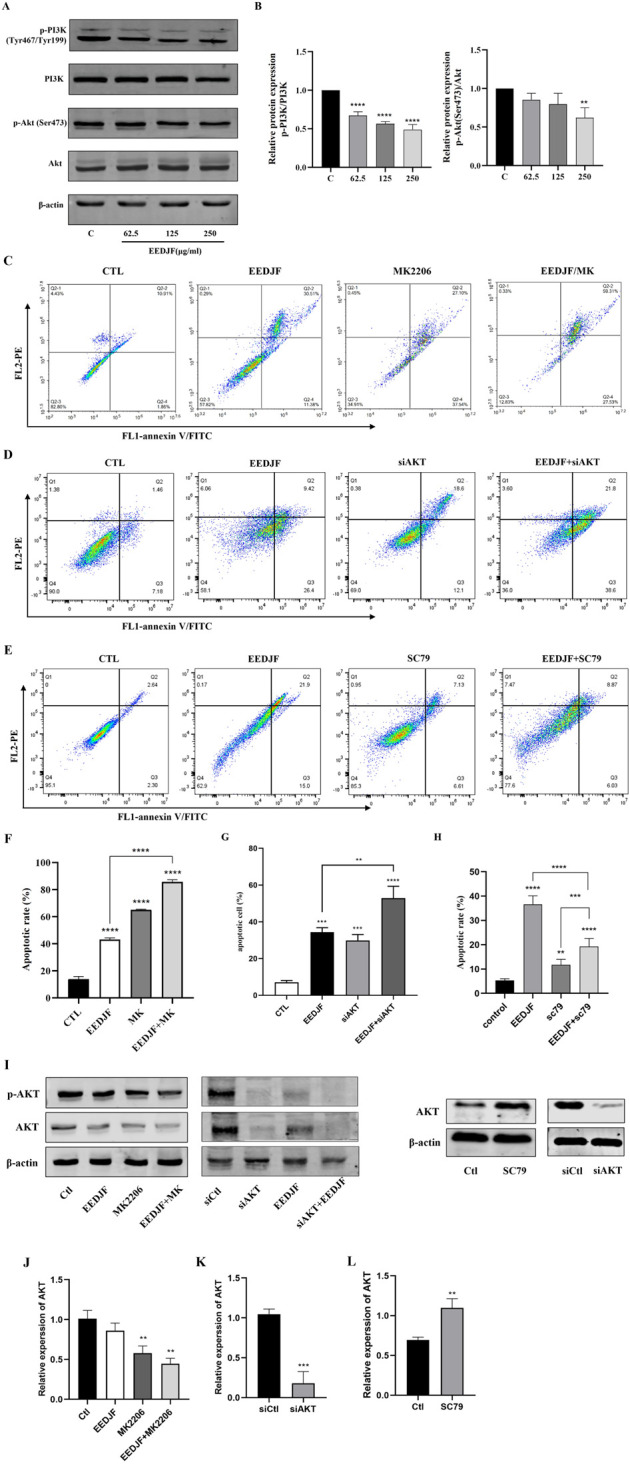
EEDJF induces apoptosis in HCT116 cells via inhibition of the PI3K–AKT signaling pathway. **(A)** Western blot analysis of phosphorylated and total PI3K and AKT following EEDJF treatment. **(B)** Quantification of relative protein expression levels shown in **(A)**. **(C–E)** Flow cytometric analysis of apoptosis in HCT116 cells treated with EEDJF alone or in combination with siAKT, MK2206, or SC79. **(F–H)** Quantitative analysis of apoptotic cell populations corresponding to **(C–E)**. **(I)** Western blot analysis of AKT expression under different treatment conditions. **(J–L)** Quantification of AKT and apoptosis-related protein expression. Band intensities were quantified using ImageJ software and normalized to β-actin. Data are presented as mean ± SD (n = 3). *p < 0.05, **p < 0.01, ***p < 0.001, ****p < 0.0001. These results demonstrate that modulation of AKT activity directly influences the sensitivity of CRC cells to EEDJF treatment.

Western blot analysis confirmed that EEDJF significantly suppressed phosphorylation of PI3K and Akt (Figures 6A,B), indicating effective inhibition of this signaling pathway. Functional validation demonstrated that Akt silencing (siRNA) or pharmacological inhibition (MK2206) significantly enhanced EEDJF-induced apoptosis (Figures 6C,D,F,G), whereas Akt activation (SC79) attenuated these effects (Figures 6E,H).

Furthermore, the changes in Akt expression (Figures 6I–L) indicate modulation of the PI3K/AKT signaling pathway. These findings suggest that EEDJF-induced apoptosis may be mediated, at least in part, through the inhibition of AKT signaling.

This correction is necessary because the original statement is not supported by any data in the figures; the corrected statement now matches the presented AKT data.

### 3.4 EEDJF induces G1/S phase arrest via PI3K/Akt/p21 signaling

**FIGURE 7:**
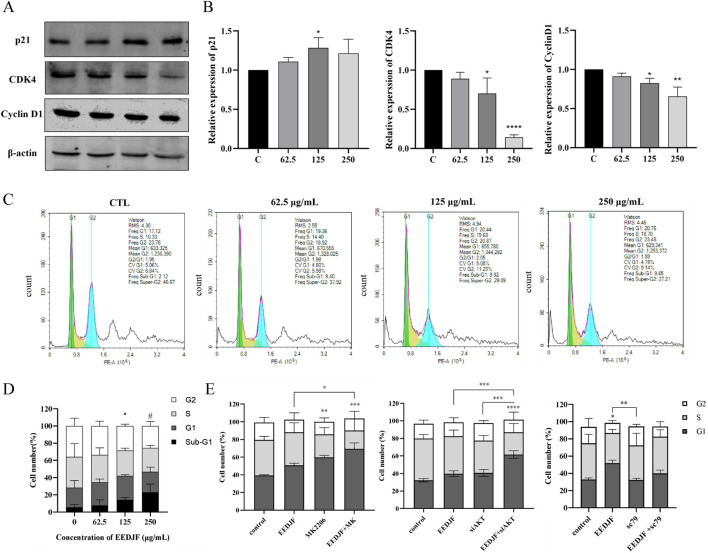
EEDJF induces G1/S cell cycle arrest in HCT116 cells. **(A)** Western blot analysis of cell cycle–related proteins (p21, Cyclin D1, and CDK4) following EEDJF treatment. **(B)** Densitometric quantification of protein expression shown in **(A)**. Band intensities were quantified using ImageJ software and normalized to β-actin. **(C)** Flow cytometric analysis of cell cycle distribution after EEDJF treatment. **(D,E)** Quantitative distribution of cells in G1, S, and G2/M phases. Data are presented as mean ± SD from three independent biological experiments. *p < 0.05, **p < 0.01, ***p < 0.001, ****p < 0.0001.

Flow cytometry revealed a significant accumulation of HCT116 cells in the G1 phase following EEDJF treatment (Figures 7A,B), indicating G1/S arrest. Western blot analysis showed upregulation of p21 and downregulation of Cyclin D1 and CDK4 (Figures 7C,D), confirming disruption of cell cycle progression.

Further validation demonstrated that Akt inhibition (siRNA or MK2206) potentiated G1 arrest, whereas Akt activation reversed this effect (Figure 7E), confirming the functional role of PI3K-Akt signaling.

Collectively, these results demonstrate that EEDJF exerts broad-spectrum anticancer activity through coordinated regulation of apoptosis and cell cycle progression via the PI3K-Akt/p21 signaling axis. Although only two CRC cell lines were evaluated, the combination of genetic (siRNA) and pharmacological (inhibitor and activator) approaches strengthens the robustness and reliability of the mechanistic conclusions.

## 4 Discussion

This study combined computational prediction, proteomic profiling, and functional assays to investigate the anti-colorectal cancer activity of EEDJF. *In vitro* experiments demonstrated that EEDJF inhibited cell proliferation in both HCT116 and SW480 colorectal cancer cell lines, supporting its reproducible antiproliferative effect across different CRC models. In contrast, EEDJF showed minimal cytotoxicity toward non-malignant HaCaT epithelial cells under the tested conditions, suggesting a relatively selective inhibitory effect on CRC cells. Integrated analyses consistently highlighted PI3K-Akt-related signaling among the enriched pathways, suggesting that this pathway may be involved in the observed biological effects. Functional modulation of Akt using siRNA, the inhibitor MK2206, and the activator SC79 further supported the involvement of Akt signaling in EEDJF-induced apoptosis and G1/S cell cycle arrest.

The PI3K-Akt pathway is frequently dysregulated in colorectal cancer and is closely associated with cell proliferation, survival, metastasis, and therapeutic resistance. In this context, the present findings suggest that EEDJF may exert its effects, at least in part, through modulation of PI3K-Akt-related signaling and downstream regulators such as p21, Cyclin D1, and CDK4. These observations are generally consistent with previous studies showing that polyphenol-rich natural products can influence CRC progression through coordinated regulation of apoptosis- and cell cycle-related pathways.

Polyphenolic constituents identified in EEDJF, including procyanidin B1 and quercetin, may contribute to the observed effects. Nevertheless, these associations are primarily based on computational prediction and prior reports, and further studies are needed to clarify their direct biological relevance in the context of the crude extract.

While the integrated multi-omics strategy provides a useful framework for exploring potential mechanisms, it should be noted that computational approaches such as network pharmacology and molecular docking are inherently predictive and mainly serve to generate hypotheses for experimental validation. In addition, EEDJF represents a complex mixture of multiple constituents. Although major compounds were characterized by LC-MS, their relative contributions, effective concentrations, and reproducibility across preparations merit further investigation. Mechanistic validation in the present study was primarily conducted in HCT116 cells, and future studies using additional CRC models and *in vivo* systems would help to further substantiate these findings and better evaluate the pharmacological safety profile of EEDJF.

Taken together, these findings indicate that EEDJF exhibits anti-CRC activity *in vitro* and provide a foundation for future work aimed at identifying active components, validating efficacy in more physiologically relevant models, and further elucidating its pharmacological potential.

## 5 Conclusion

In summary, EEDJF inhibited proliferation and induced apoptosis and G1/S cell cycle arrest in CRC cells, while showing minimal cytotoxicity toward HaCaT epithelial cells under the tested conditions. Integrated computational and experimental analyses suggest that these effects may be associated, at least in part, with modulation of PI3K-Akt/p21 signaling. These findings support further investigation of EEDJF as a potential source of bioactive compounds for colorectal cancer research.

## Data Availability

The original data generated in this study are included in the article and its Supplementary Material. Further inquiries can be directed to the corresponding author.
